# Anatomical and Functional Impacts of Congenital Bilateral Visual Deprivation on the Visual Pathway—A Comprehensive Review

**DOI:** 10.3390/jcm13061775

**Published:** 2024-03-20

**Authors:** Aleksandra Czarnek-Chudzik, Mario Damiano Toro, Robert Rejdak, Katarzyna Nowomiejska

**Affiliations:** 1 Chair and Department of General and Pediatric Ophthalmology, Medical University of Lublin, ul. Chmielna 1, 20-079 Lublin, Poland; olaczarnek@poczta.fm (A.C.-C.); robertrejdak@yahoo.com (R.R.); 2 Public Health Department, University of Naples Federico II, 80138 Naples, Italy; toro.mario@email.it

**Keywords:** inherited retinal diseases, congenital visual deprivation, bilateral visual deprivation, magnetic resonance imaging

## Abstract

Neuroplasticity is a complex process that is heightened during time-sensitive periods of pre- and postnatal brain development. It continues, albeit to a lesser extent, throughout adolescence and young adulthood. Congenital visual deprivation is well-known and explored in human-model behavioral research. In this study, we review existing research on neuroadaptations and neuroplasticity of the visual pathway as a result of inherited retinal diseases (IRD), focusing on data concerning congenital bilateral visual deprivation in humans published in PubMed in the past 5 years, including 18 articles. We highlight evidence about the anatomical and behavioral aspects of neuroplasticity as different brain responses to different types of visual deprivation. We also focus on various very interesting aspects of the cross-modal functional reorganization of the visual and auditory cortex as an example of brain plasticity due to combined visual and auditory loss. Our study shows that central nervous system magnetic resonance imaging (MRI) advancements have allowed researchers to report previously elusive anatomical evidence. Patients with a known mechanism of IRD—examined with high magnetic field MRI and functional MRI—have been proven to be adequate models to explore neuroadaptations of the visual pathway due to bilateral, early, and late visual deprivation.

## 1. Introduction

Neuroplasticity refers to the inherently dynamic biological capacity of the central nervous system (CNS) to undergo maturation, change structurally and functionally in response to experience, and adapt following injury [1,2]. The brain has a lifelong ability to modify and adapt, not only as a consequence of pathology but also in response to behavioral and environmental adaptations. The capacity for brain reorganization varies across different sensory and cognitive modalities, and some neural functions demonstrate little or no ability to adapt after critical periods in development [3].

The visual system is unique in a way that a great part of visual processing occurs outside the brain within the retina of the eye. Central vision is recorded at the retinal level by the macula, which covers about 20° of the central visual field and provides high spatial resolution. The peripheral visual field is recorded by the rest of the retina and has low spatial resolution. The central (macula) and peripheral retinas form a complex functional unit. Their combined contribution is responsible for the impression of a homogeneous image across the visual field. This functional unity is not present from birth because, ontogenically, the central and peripheral parts of the retina mature sequentially. In contrast to the periphery, the central retina is immature at birth and develops fully only after several years. How the brain behaves and potentially adapts to impaired central or peripheral vision remains unclear.

The distinction between central and peripheral vision is maintained within the brain. Approximately 50% of the primary visual cortex is devoted to the central visual field [4]. Dorsal visual areas receive relatively more projections from the areas processing peripheral visual field representations, whereas ventral visual areas are more densely connected to those processing central representations. Recent evidence points to white matter plasticity.

With the development of neuroimaging methods, two types of connections in the brain—structural and functional—have been distinguished [5]. Vision loss seems to require a substantial remodeling of the anatomical and functional neural networks of the entire brain since sight dominates the human sensorium and because the stimulus is presented in multiple sensory modality inputs [6,7]. Researchers began to analyze the brain as a complex network in order to better understand global brain organization and explain neurophysiological processes such as inter-modular integration or cognition [8].

Structural brain connectivity has been observed in multiple ophthalmic diseases. Recent studies in glaucoma patients have shown that pre-geniculate (optic tract and chiasm), geniculate, and post-geniculate (the optic radiation (OR) and primary visual cortex) structures are affected in glaucoma as well, at least at later stages of the disease. Moreover, changes in white matter (WM) tracts have been observed beyond primary visual pathways, such as the anterior thalamic radiation, corticospinal tract, superior longitudinal fasciculus, and forceps major. WM volume abnormalities have also been observed in Leber’s hereditary optic neuropathy, high myopia, and optic neuritis. In the case of retinitis pigmentosa (RP) subjects, an analysis of structural connections based on recording brain fluid movement (such as the magnetic resonance diffusion tensor (DTI) test) has shown WM matter fiber atrophy in different areas of the optic tract. It has been already observed that the plastic potential is higher for congenital than for acquired visual pathway defects [9,10].

Inherited retinal diseases (IRD) have a genetic cause and often lead to progressive visual loss or blindness. While all IRD fit the European definition of rare (i.e., affecting </=5 in 10,000 people), they are a common cause of visual impairment registration in the working-age population and the leading cause of visual impairment and loss in individuals aged between 15 and 45. 

There is still an ongoing debate on the nature of adult brain functional reorganization induced by retinal diseases [3,11,12]. There is a lack of knowledge about visual pathway changes in advanced visual field loss. In addition, little is known about the anatomical consequences and compensation mechanisms occurring after central or peripheral visual deprivation. Kitajima and colleagues have found that the calcarine fissure increases in width in RP patients, notably within anterior and middle regions that normally represent peripheral vision. This suits the progression of visual deprivation in RP, which begins from the peripheral visual field and narrows towards the fovea [13].

### Magnetic Resonance Imaging

Currently, 7 Tesla MRI and functional magnetic resonance imaging (fMRI) scanners are the best tools to learn more about anatomical and functional changes in the central nervous system. These non-invasive, high-resolution imaging technologies are used to assess anatomical brain structures, detect neuroactivity, and visualize brain function.

Magnetic resonance imaging (MRI) has been used in previous ophthalmological studies to analyze the visual pathway, including performing morphologic examinations and functional measurements. Using MRI, researchers can non-invasively examine the effects of eye diseases on the whole visual pathway, including the lateral geniculate nucleus and striate and extrastriate cortices. Over the last few decades, advances in MRI technology have allowed for higher-resolution images to be captured, with better tissue contrast and spatial resolutions of <1 mm.

Structural MRI has revealed abnormalities in gray and white matter density across both visual and non-visual areas in conditions including amblyopia, albinism, glaucoma, and macular degeneration. Although there have been relatively few studies on RP, they have shown deterioration within the anterior and middle occipital lobes but have not revealed any abnormalities earlier within the visual pathway. Therefore, the conclusion is that the visual cortex undergoes changes following the loss of functional inputs. It remains to be discovered whether these changes are permanent and irreversible—this could have implications for visual-restoration treatments. Existing MRI methods (1.5 Tesla and 3 Tesla MRI) are still unlikely to illuminate these issues, but it is important to continue research in this area. In addition to helping explain the way in which different diseases affect the visual pathway—from the retina through to the visual cortex—ultrahigh-field 7 Tesla MRI could be used to aid in diagnosis and determine the potential course and efficacy of treatments. 7 Tesla MRI provides high isotropic resolution (<0.8 mm) and excellent gray/white matter contrast of MT-Silent to facilitate the visualization of small structures in the brain. 7 Tesla MRI scanners have improved both spatial resolution and tissue signal, allowing the visualization of anatomical elements that are undetectable at lower field strengths. Brain scans using this technology have already been used in glaucoma and Leber hereditary optic neuropathy (LHON). Some authors have concluded that in the course of glaucoma, the lateral geniculate body (LGB) volume decreases [14]. These changes are correlated with the thickness of the retinal nerve fiber layer (RNFL) in the early stages of glaucoma and are not correlated with visual field indices. LHON patients showed a significantly decreased volume of the right LGB and the right medial geniculate nuclei. The volume of the right lateral geniculate nucleus (LGN) was strongly correlated with the average thickness of the right RNFL. The abnormalities in the volume of LHON patients’ thalamic nuclei indicate that the disease can cause changes not only in the white matter areas constituting visual tracts but also in gray matter structures.

fMRI was invented in the early 1990s and has since played a vital role in understanding the brain. It has been widely used in cognitive neuroscience and studies of mental diseases because of its superior temporal and spatial resolution [15]. fMRI may therefore be useful for evaluating residual visual pathway function in IRD [16].

fMRI has rapidly become the standard technique for inferring neuronal activity in human subjects because it is non-invasive and provides reliable response localization, as well as superior spatial resolution compared to older technologies such as positron emission tomography (PET).

Although there is currently no cure for the majority of retinal dystrophies, recent treatments focus on restoring visual function via a variety of approaches. However, the success of retinal restoration depends on the extent to which the rest of the visual pathway function and structure have remained stable from the point that sight was lost, without any significant degeneration or reorganization.

The aim of this study was to search for evidence of neuroplasticity in response to visual deprivation due to congenital vision loss in IRD.

## 2. Materials and Methods

**Figure 1:**
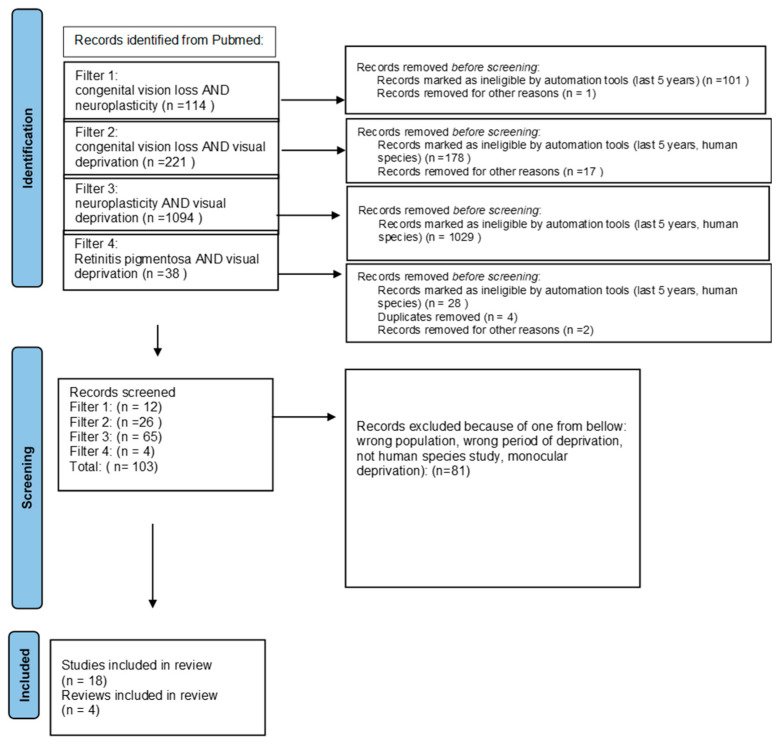
PRISMA 2020 [17] flow diagram.

We ran a PubMed search using 4 types of filters: congenital vision loss AND neuroplasticity, congenital vision loss AND visual deprivation, neuroplasticity AND visual deprivation, and retinitis pigmentosa AND visual deprivation in order to find publications about the anatomical and behavioral influence of visual deprivation caused by congenital vision loss. We used automatic tools—‘last 5 years’ and ‘human species’—to limit information to the newest and excluded monocular deprivation. We expanded the information obtained through the search with some previously published data about rare inherited retinal diseases. We used the PRISMA guidelines for searching for the keywords [17] (Figure 1).

## 3. Results

### 3.1. Development of Brain Neuroplasticity

Developmental neuroplasticity is a blanket term used to refer to fundamental changes in neurogenesis, neuronal cell migration, synapse formation, and structural and functional neuronal network specialization, which lead to the behavioral acquisition of motor and non-motor developmental milestones, as well as adaptation to a constantly changing environment through learning and memory. Several mechanisms contribute to the intricate balance between neural plasticity and homeostasis in the developing brain. This allows a reasonable degree of synaptic function (not too rigid and not too flexible) and network stability [9].

Patterns of abnormal neuroplasticity have been recently recognized as core pathologies in many congenital and acquired pediatric disorders of the central nervous system (CNS) such as neonatal hypoxic-ischemic encephalopathy, cerebral palsy, epilepsy, epileptic encephalopathies, dystonia, intellectual disabilities, autism spectrum disorders (ASD), and neuropsychiatric disorders such as attention deficit hyperactivity disorder (ADHD) and schizophrenia. Hence, the potential of modulating abnormal plasticity patterns in childhood disorders of the brain is gaining momentum in translational pediatric neuromodulation research [10].

Some patterns of neuroplasticity expressed by the developing brain have been suggested: (1) developmental plasticity, which is further classified into normal and impaired developmental plasticity as seen in syndromic autism spectrum disorders, (2) adaptive (experience-dependent) plasticity following intense motor skill training, (3) reactive plasticity to pre- and post-natal CNS injury or sensory deprivation, (4) excessive plasticity (loss of homeostatic regulation) as seen in dystonia and epilepsy, (5) and finally, plasticity as the brain’s “Achilles tendon”, inducing brain vulnerability under certain conditions such as hypoxic ischemic encephalopathy and epileptic encephalopathy syndromes [1].

The brain’s unique capacity to rearrange emerging or already existing networks is very complex and involves different levels of the central nervous system. From a biological perspective, neurogenesis, synaptogenesis, and synaptic pruning represent the building blocks for CNS plasticity. These biological processes are subjected to genetically programmed, time-limited periods. These periods, known as “critical” or “sensitive”, are periods during which the brain is the most amenable to change [11]. Neurogenesis is the most prominent during early fetal development. This is followed by robust synaptogenesis that starts as early as 27 weeks after the gestational age and intensifies over the first two years of life following a heterochronous cortex-specific maturational pattern [12].

On a molecular level, CNS receptors undergo major alterations to achieve adult-like patterns and are thought to regulate the critical and sensitive periods of development. Receptors that respond to the neurotransmitter gamma-aminobutyric acid (GABAA), the *N*-methyl-D-aspartate receptor (NMDA)—a receptor of glutamate, the primary excitatory neurotransmitter in the human brain—and the α-amino-3-hydroxy-5-methyl-4-isoxazolepropionic acid (AMPA) receptor, an ionotropic transmembrane receptor for glutamate that mediates fast synaptic transmission in CNS) receptors, follow a sequential expression in the developing brain that subsequently allows GABAergic interneurons to develop, mature, connect, and generate patterns before pyramidal cells [13]. Similarly, NMDA receptors develop before AMPA receptors, providing the basic platform for neuronal communication and network formation. Finally, AMPA receptors develop at the appropriate time for experience-dependent long-term potentiation (LTP) and long-term depression (LTD) plasticity. This sequential expression is not mutually exclusive. Moreover, crucial developmental alterations occur on a CNS-wide scale, with glutamate and GABA receptors switching their subunit composition to trigger a cascade of intracellular and synaptic events that renders the developing brain more susceptible to perturbations.

The embryonic brain abounds with the two major types of glutamate receptor subunits, GluN2B and GluN2D. The composition of NMDAR subunits undergoes modifications during the first weeks of life. The prenatally dominant GluN2B subunit switches to the GluN2A subunit during a time window that coincides physiologically with the critical periods for synaptic maturation and changes in electrophysiological properties leading to circuit formation and the acquisition of learning abilities. This developmental switch curbs premature synaptic maturation, suggesting its critical role in neural activation and experience-dependent synaptic plasticity [14].

Structurally, age- and sex-related changes in brain volume relative to head circumference, cortical thickness, and surface area and in cortical, subcortical, and cerebellar anatomy have been extensively described [15]. The dynamic mapping of human cortical development has been postulated to temporally reflect changes at the level of synaptogenesis and synaptic pruning [16].

fMRI studies have identified multiple functionally specialized regions in the ventral visual pathway that respond selectively to particular object categories (e.g., faces, words, and general objects) [17]. Although adult-like object-selective responses are observed in children as early as the age of five [18,19], object selectivity reaches adult levels only in adolescence [20,21].

Enhanced plasticity occurs in the peak phases of physical growth and may therefore allow for continuous perception during the expansion of the body surface. For example, visual receptive fields must repeatedly remap as the distance between the two eyes increases. Indeed, experience-dependent matching of stimulus selectivity of the visual input from the two eyes occurs during the critical period [22]. Changes in the microstructure of white matter tracts have been found in late-blind subjects when compared to the congenitally blind or sighted controls [23,24,25].

### 3.2. Early Onset of Congenital Visual Deprivation

Early onset of deprivation should have the biggest damaging influence on a developing brain. Dennis et al. suggested that recovery scenarios may vary depending on time and “starting” state. Early-onset disorders have no period of normal brain and behavioral development against which age expectations can be referenced; so, this leads to questions concerning how well dysmorphic substrates can support skill development. Later onset conditions, varying with age at onset and time since injury, concern how brain insult affects pre-injury skill maintenance, as well as ongoing skill development [10]. Normal visual development requires unimpeded and coordinated input from each eye to the visual cortex during an early critical period of cortical maturation. Disrupted binocular vision during this critical period, due to visual deprivation (e.g., congenital cataract), can lead to a neurodevelopmental disorder of vision [18,26]. This in turn leads to neuroadaptations in both the reception and analysis of stimuli provided by the surrounding environment. Studies analyzed in our review showed vivid differences in adaptation in congenitally blind children and controls based on behavioral experiments. 

The first one—provided by a research group led by Topalidis—asked whether spatial selective attention is necessary for the processing of affective prosodies—sound properties of speech such as accent or intonation, which give language an emotional meaning—after visual deprivation from birth. For this purpose, pseudowords spoken in happy, neutral, fearful, or threatening prosodies were presented at the left or right loudspeaker. Congenitally blind individuals (*n* = 8) and sighted controls (*n* = 13) had to attend to one of the loudspeakers and detect rare pseudowords presented at the attended loudspeaker during EEG recording. The results provide evidence for “emotion-general“ auditory spatial selective attention effects in congenitally blind individuals and suggest a potential reorganization of the voice processing brain system following visual deprivation from birth [19].

In another experiment, Setti and coworkers investigated how spatial working memory skills, as well as the processing and retrieval of distal auditory spatial information, are influenced by visual experience. They developed an experimental paradigm using an acoustic simulation. The performance of congenitally blind and sighted participants (*n* = 9 per group) was compared when recalling sequences of spatialized auditory items in the same or reverse order of presentation. Two experimental conditions based on stimuli features were tested: non-semantic and semantic. Blind participants had a shorter memory span in the backward than the forward order of presentation. In contrast, sighted participants did not respond similarly, suggesting that blindness affects spatial information processing with greater executive source involvement. The results suggest that a lack of early visual experience affects the ability to encode the surrounding space [20].

As demonstrated by the study performed by Cappagli et al., the ability to represent positive and negative numbers in horizontal and sagittal planes in visually impaired children is also disturbed. They tested 13 children with low vision and 8 children who were completely blind in their experiment. They adopted the number-to-position paradigm by Crollen et al. [21], asking children to indicate the spatial position of positive and negative numbers on a graduated rule positioned horizontally or sagittally in the frontal plane. The results suggest that long-term visual deprivation alters the ability to identify the spatial position of numbers independently of the spatial plane and the number polarity. Moreover, the results indicate that relying on poor visual acuity is detrimental for low-vision children when asked to localize both positive and negative numbers in space, suggesting that visual experience might have a differential role in numerical processing depending on number polarity. Such findings increase the knowledge related to the impact of visual experience on numerical processing. Since both positive and negative numbers are fundamental aspects of learning mathematical principles, the outcomes of the present study provide information about the need to implement early rehabilitation strategies to prevent the risk of numerical difficulties in visually impaired children [22].

Rimmele and colleagues went a step further in their experiments, collecting data comparing the activity of the brain before and after special training. They used an auditory working memory training paradigm in congenitally blind and sighted adults. They hypothesized that the visual cortex integrates into auditory working memory networks after they have been challenged by training. The spectral profile of functional networks, which mediate cross-modal reorganization following visual deprivation was investigated. A training-induced integration of the visual cortex into task-related networks in congenitally blind individuals was expected to result in changes in long-range functional connectivity in the theta-, beta-, and gamma-bands (imaginary coherency) between the visual cortex and working memory networks. Magnetoencephalographic data were recorded in congenitally blind and sighted individuals during the resting state as well as during a voice-based working memory task; the task was performed before and after working memory training with either auditory or tactile stimuli or with a control condition. Auditory working memory training strengthened theta-band (2.5–5 Hz) connectivity in the sighted participants and the beta-band (17.5–22.5 Hz) connectivity in the blind participants. In sighted participants, theta-band connectivity increased between brain areas typically involved in auditory working memory (inferior frontal, superior temporal, and insular cortex). In blind participants, beta-band networks largely emerged during the training, and connectivity increased between brain areas involved in auditory working memory and, as predicted, in the visual cortex. It showed long-range connectivity as a key mechanism of functional reorganization following congenital blindness and provided new insights into the spectral characteristics of functional network connectivity [23].

In a different study, Lin and colleagues provided deeper insights into brain structures by investigating four groups of subjects: congenitally blind children (*n* = 8), congenitally blind adults (*n* = 22), sighted children (*n* = 10), and sighted adults (*n* = 21). They performed structural 3D images, resting-state functional magnetic resonance imaging (rs-fMRI) images, and behavioral performance data for multiple cognitive tasks for each subject. They collected three analyses: (1) regional gray matter volume (GMV) analysis in the thalamus to identify the vision/age-relevant thalamic regions, (2) functional connectivity (FC) analysis between the thalamus and cerebral cortex to identify the vision/age-relevant FCs with the vision/age-relevant thalamic regions, and (3) nodal GMV analysis in the cerebral cortex to identify the other vision/age-relevant cortical regions that were end nodes of the vision/age-relevant FCs. 

The conclusions from the results are that blindness modulates and changes the structural development of the visual and somatosensory thalamus and elicits the somatosensory thalamic network to process tactile information [24].

Additional research with congenitally blind adults confirmed these findings. Magnetoencephalography was also used to collect the data of congenitally blind and sighted humans in another study [25]. Deprivation-related changes in spectral profiles were mapped to the cortex using clustering and classification procedures. Altered spectral profiles in visual areas suggest changes in visual alpha–gamma band inhibitory–excitatory circuits. Remarkably, spectral profiles were also altered in auditory and right frontal areas, showing increased power in theta-to-beta frequency bands in blind compared to sighted individuals, possibly related to adaptive auditory and higher cognitive processing. Moreover, there was a correlation between occipital alpha and microstructural white matter properties extending bilaterally across posterior parts of the brain. These data show that visual deprivation selectively modulates spectral profiles, possibly reflecting structural and functional adaptation [26].

After neuroimaging testing 15 congenitally blind participants, Rączy and colleagues highlighted that ventral occipitotemporal cortex and auditory priming in general language areas in the blind serve multiple functions, one of which, orthographic processing, overlaps with its function in the sighted participants. 

Very complex studies aimed to check cognitive possibilities and examine the brain areas that were recently targeted. Chebat and coworkers performed a study to correlate the performance of congenitally blind individuals (CB) and blindfolded sighted controls (SC) in a life-size obstacle course using a visual-to-tactile sensory substitution device, with the size of brain structures (voxel-based morphometry—VBM) measured through structural magnetic resonance imaging (MRI). The results show that CB has similar learning capabilities as SC for the detection (LRD) and avoidance (LRA) of obstacles [27].

Arbel and colleagues characterized the temporal dynamics of the development of neuronal specialization in a deprived visual cortex. They tested the effect of an unusual sensory input (in neural specialization new spelling, OVAL) through an unusual auditory modality in congenitally blind sensory-substitution-device (SSD) users. The results show that while after 2 h of SSD training, we can already observe the recruitment of the deprived ventral visual stream by auditory stimuli, computation-selective cross-modal recruitment requires longer training to establish [28].

Other brain regions apparently unrelated to the visual pathway have become scientists’ targets as potential sites of neuroadaptation due to visual deprivation. Chouinard-Leclaire et al. compared cortical morphology and olfactory bulb volume in congenitally blind and normally sighted individuals. The results show that the absence of visual input leads to morphological alterations in olfactory processing areas because blind individuals exhibited smaller olfactory bulbs and alterations of cortical density in some higher olfactory processing centers but unchanged cortical thickness [29].

Because of its reversibility, the congenital cataract is one of the most extensively studied human models of visual deprivation [25]. Depending on the age at which surgery is performed, it provides a good way of observing changes in the brain before and after surgery [30]. Senna and colleagues tested participants who were surgically treated for congenital dense bilateral cataracts several years after birth. In Experiment 1, they assessed the participant’s ability to understand spatial relationships between sounds by asking them to spatially bisect three consecutive, laterally separated sounds. The participants performed better before surgery compared to after surgery. Nonetheless, they performed worse than sighted controls. In Experiment 2, they demonstrated that single sound localization in the two-dimensional frontal plane improves quickly after surgery, approaching performance levels of sighted controls. Such recovery seems to be mediated by visual acuity as participants gaining higher post-surgical visual acuity performed better in both experiments. These findings provide significant support for the hypothesis that vision calibrates auditory space perception. Importantly, this also demonstrates that this process can occur even when vision is restored after years of visual deprivation [31]. Furthermore, various research groups from Canada, Belgium, and Italy showed that cataract-reversal patients give enhanced salience to auditory stimuli, as measured by faster reaction times on auditory trials and faster switches from vision to audition when compared to controls. This enhanced speed at detecting auditory targets is reminiscent of the one found after early-acquired and permanent blindness in humans [32]. Based on these data, De Heering and al. suggest that the absence of visual inputs for several months after birth induces a change in the competitive balance between the visual and the auditory modality that impacts the detection of even simple targets. Similar findings have been observed for cochlear-implanted deaf patients whose superior multisensory integration capabilities compared to controls have been linked to their enhanced visual abilities, especially in noisy situations [33]. These findings could also be considered the psychophysical counterpart of the observations that, like early-blind individuals [34], cataract-reversal patients show enhanced reactivity in response to sounds in regions that typically process vision [35] and, unlike controls, show lower visual cortical activity during audiovisual stimulation than during visual stimulation alone [36].

Scientists from Israel approached the subject in a different way. Zohary and co-authors examined children who suffered from early-onset bilateral cataracts and were diagnosed and operated on many years later (at age 11.3 ± 3.3 y). Typically, following surgery, the children showed dramatic improvement in their visual acuity, although it was still very poor with respect to normal visual acuity. The study also included 11 Israeli children (age 10.1 ± 3.3 y) with congenital cataracts that were surgically treated within a few months after birth. These children typically have only a slight loss of visual acuity [37,38,39]. They used the gaze-cueing paradigm in two experiments and measured the effect of a gaze cue generated by a change in the eye position or head orientation. In both experiments, at the beginning of the trial, participants were required to touch the nose of the seen face, thereby ensuring that they focused their attention on the face and fixated near the actor’s eyes (∼2°), without obstructing the eyes themselves. Touching the nose led to a presentation of the gaze cue (eyes or head shifting to the left or right, perceived as apparent motion). Then, 300 ms later, a balloon appeared either in agreement with the gaze direction (“compatible”) or on the opposite side (“incompatible”). Participants were instructed to touch the balloon as quickly as possible, and the reaction time was monitored. After surgery, the patient’s visual acuity improved substantially, but the patients failed to show eye gaze-following effects and fixated less than controls on the eyes, two spontaneous behaviors typically seen in controls. This model for learning gaze direction explains how head-based gaze-following can develop under severe image blur, resembling preoperative conditions. It also suggests why automatic eye gaze-following is not established after surgery due to a lack of detailed early visual experience. This means that visual skills acquired in infancy in an unsupervised manner will be difficult or impossible to acquire when internal guidance is no longer available, even when sufficient image resolution for the task is restored. This causes fundamental limitations to spontaneous vision recovery following prolonged deprivation at an early age [37].

The first one examined adult cataract-reversal patients who were deprived of any patterned visual input from birth for a period of 2–9 months. They found increased cortical thickness in the occipital cortex, including the calcarine sulcus in both hemispheres, where the primary visual cortex (V1) is concentrated, as well as the occipital pole in the right hemisphere [40]. Guerreiro and colleagues obtained similar results. In the group of individuals with a history of cataracts, cataract surgery took place between the ages of 5 and 24 months. They also reported increased cortical thickness in the occipital cortex, including the left calcarine sulcus [41]. These results show that visual experience in the first several months of life influences cortical thickening and thinning in the occipital lobe. 

### 3.3. Late Onset of Congenital Visual Deprivation

#### 3.3.1. Retinitis Pigmentosa 

Retinitis pigmentosa (RP) is a group of severe hereditary diseases of the retina that lead to progressive impairment of photoreceptor cells. Mutations in 1 of over 150 causal genes identified to date lead to the classical clinical appearance of bone spicule formation in the peripheral retina, blood vessel attenuation, and optic disc pallor. In accordance with the large number of gene defects associated with RP, the individual clinical findings and the progression of gene-specific subtypes of RP are very heterogeneous and variable [42]. RP is the most common form of inherited retinopathy, with a prevalence of approximately 1 in 3500. RP disease is rod-predominant photoreceptor degeneration, with late-stage atrophy of the cones and retinal pigment epithelium. It presents early in life, with nyctalopia as a common first symptom. In the advanced stage, it leads to blindness. The majority of RP cases are non-syndromic—i.e., isolated to the eye. Currently, there are no proven therapeutic options to improve or halt the slowly progressive visual loss that occurs in patients with RP. Available treatments include pharmacological approaches, gene therapy, cell transplantation, retinal prostheses, and optogenetics [43]. Gene therapy is the only promising strategy. However, it is restricted to small groups of patients. All new therapeutic strategies are focused on preserving existing photoreceptors or substituting light-responsive elements. 

Although the retinal structure and optic pathway are substantially preserved, at least in the early stages of RP, studies describing the condition of the visual pathway in RP are in short supply. Because of the declining state of the underlying disease—which is total blindness—psychophysical implications for RP patients are the same as those for blind patients, but for different reasons. In essence, vision loss in blind and RP patients causes other senses to sharpen in a similar way [44,45,46].

Since the advent of advanced neuroimaging, there have been growing data showing the restorative impact of visual deprivation on the brain. In 2019, Dan et al. presented the results of 3 tesla MRI scans showing reduced synchronicity of V1 neural activities in individuals with RP. Moreover, RP individuals exhibited intrinsic visual network disconnection and reorganization of the retino-thalamocortical pathway and dorsal visual stream, suggesting impaired visuospatial and stereoscopic vision [47]. Some studies have shown that resting-state functional connectivity in RP is lower than normal and that the synchronicity of neural activity changes in the primary visual area is reduced in RP individuals. One study showed significantly decreased gray matter volume (GMV) in V1 in RP individuals. The decreased GMV values were closely linked to the degree of visual field deficit [13]. In a study by Hoffsteter and colleagues, the brains of two patient groups suffering from advanced retinitis pigmentosa with a specific deterioration of the visual field were examined using 3 Tesla MRI [48]. One group had lost their peripheral visual field, retaining only central (“tunnel”) vision, while the other consisted of blind patients with complete visual field loss. They showed gradual changes in diffusivity, indicative of degenerative processes in the primary visual pathway comprising the optic tract and the optic radiation. Moreover, changes were also found in tracts of the ventral stream and the corticospinal fasciculus. There was a gradual reorganization of these tracts as a consequence of the gradual loss of visual field coverage (from intact perception to partial vision to complete blindness). Some authors have also observed a decrease in diffusion anisotropy in the visual pathways, both anterior and posterior to the relay station, in the lateral geniculate body.

#### 3.3.2. Usher Syndrome

Usher syndrome (USH) is a very interesting model of visual deprivation. It is a rare autosomal recessive disease that combines IRD and sensorineural hearing loss (±vestibular dysfunction). It is the most common syndromic IRD, with a prevalence ranging from 3 to 6.2 per 100,000. The majority of patients with USH usually fall into one of three clinical categories. Of these, Usher syndrome type I (USH1) is the most severe form, characterized by profound hearing loss and vestibular dysfunction from birth. Moreover, the onset of IRD occurs earlier in USH1 than in Usher syndrome type II (USH2), which produces less severe congenital hearing loss and does not impair normal vestibular function. Usher syndrome type III (USH3) is the least common form. This ciliopathy caused by various mutations of the usherin gene leads to vision and hearing loss. Auditory deprivation (hearing loss) in this disease is partially eliminated through the use of cochlear implants. Depending on the awareness of the population and financial possibilities, the use of cochlear implants—an option that is not always known and financially available, depending on the population—can reduce hearing loss to some extent. Visual impairment, however, remains incurable in this disease. Inner ear cells and retinal photoreceptor cells are known to be affected in USH and have been linked to cilia defects [49]. Since olfactory receptor cells are ciliated, the hypothesis of olfactory loss in USH has emerged [50]. Some behavioral reports identified evidence of olfactory dysfunction in USH patients during odorant identification and detection tests [51]. Moreover, psychophysical assessments have revealed a consistent olfactory loss in USH [50]. A study on brain structural integrity also found that the olfactory sulcus was shallower in USH patients compared to the control group [52,53].

There have been reports of focal and diffuse atrophic changes in the supratentorial brain, as well as atrophy of some of the structures of the posterior fossa. Quantitative analysis was performed on MRI scans of 19 Usher syndrome patients (12 with type I and 7 with type II) looking at the cerebellum and various cerebellar components. The scans revealed cerebellar atrophy in both types and sparing of cerebellar vermis lobules I–V in type II Usher syndrome patients only. There was a significant decrease in the intracranial volume and brain and cerebellum size, with a trend towards an increase in the size of the subarachnoid spaces. These data suggest that the process of the disease in Usher syndrome involves the entire brain and is not limited to the posterior fossa or auditory and visual systems [54]. Some recent fMRI results also indicate that early visual deprivation causes the reorganization of binaural spatial processing in the auditory cortex and that blind individuals may rely on alternative mechanisms for processing azimuth position [55].

#### 3.3.3. Stargardt Disease

Stargardt disease (STGD) is the most frequent form of juvenile hereditary macular degeneration (MD). The most common form of this disease is transmitted in an autosomal recessive manner and is related to ABCA4 gene mutations. STGD is the leading type of inherited macular disease with an approximate incidence of 1 in 10,000. STGD induces well-circumscribed, central visual defects. In its advanced stages, patients affected by this hereditary cone-rod dystrophy end up losing macular vision and can rely solely on their residual peripheral vision in daily life. In a study by Melillo and colleagues, twenty-four STGD patients underwent fMRI to assess cerebral activation during visual stimulation by a flickering checkerboard in four primary visual cortex (PVC) subdivisions [56]. As a result, higher ERG responses were significantly (*p* < 0.0125) associated with larger functional cerebral responses in V1, V2, and V3 subdivisions. Cortical thickness (CoTks) and resting-state cortical entropy (rs-CoEn) obtained using 3 Tesla MRI scans were extracted in 12 STGD, 12 RP (tunnel vision stage), and 14 normally sighted subjects in a study published by Sanda and colleagues. Compared to controls, both groups with visual loss exhibited decreased CoTks in the V3d dorsal area. Peripheral visual field loss also showed a specific decrease in CoTks in the early visual cortex and V4 ventral area, while central visual field loss exhibited a decrease in CoTks in the V3A dorsal area V3A. Only central visual field loss exhibited increased CoEn in the LO-2 area and FG1 [57].

### 3.4. Early Onset and Late Onset of Blindness

Some researchers also conducted a comparison study of blind participants, among whom 22 were congenitally blind (CB), 14 were LB—who started to lose their vision later in life—and 29 were normally sighted controls (SC). Pitito and coworkers tried to observe the integrity of the retino-fugal system by measuring the optic nerve, optic chiasm, and optic tract using structural T1-weighted MRI images. The results show that the optic nerve, optic tract, optic chiasm, and LGN were reduced by 50 to 60% in CB and LB. In LB, optic nerve volume correlated negatively with blindness duration [58]. Recent data suggest that the two main components of the commissural system undergo neuroplastic changes, irrespective of the age of onset of blindness, although the alterations observed in AC are more important in congenital than late-onset blindness. These suggestions are based on MRI data. The data were acquired using a 3T scanner and included structural brain scans, resting-state fMRI, and diffusion-weighted imaging scans performed on 12 congenitally blind (CB), 15 late-blind (LB, meaning onset of blindness of 16.6 ± 8.9 years), and 15 matched normally sighted controls (SC). This supports the findings that the splenium—a structure primarily composed of fibers connecting the visual areas of the brain—is indeed sensitive to visual deprivation in both CB and LB. These data provide new insights into neuroplastic alterations of the commissural fiber system following blindness [59]. 

A summary of the major studies reviewed in this article is provided in the table below (Table 1).

## 4. Discussion

In our review, we have shown the trends in recent research and demonstrated that anatomical changes in the brain are a more common response to visual deprivation than behavioral adaptations. We focused mostly on bilateral visual deprivation caused by congenital blindness, which means it is present at birth and late onset blindnessoccuring during the development of eye. It is already known that aspects of vision that mature later during normal development are more affected by early visual deprivation than aspects of vision that mature earlier. However, it is assumed that the plastic potential is higher for congenital than acquired visual pathway defects. Interestingly, deficits in the deprived eye are greater after early monocular deprivation than after early binocular deprivation [9,10]. 

In our study, we identified a relatively large number of new publications focused on changes in the visual pathway, especially in visual cortex size due to visual deprivation [18]. Most of the studies on the anatomical changes in the brain following visual deprivation have focused on the reorganization of the visual cortex and its afferent and efferent projections [18].

Both congenital and late blindness affect the integrity of the brain’s visual structures. Atrophy is found in all the components of the retino-fugal system as there is reduced optic nerve, optic tract, optic chiasm, and LGN volume both in congenital and late blindness [58]. There are also degenerative processes in the primary visual pathway comprising the optic tract and the optic radiation in late blindness [48]. The primary visual system, including the LGN and visual cortical areas V1 to V5, shows significant volume reductions [58]. There is also lower gray matter volume in the thalamic regions of congenital blindness [24]. In contrast, individuals with congenital blindness exhibited smaller olfactory bulbs and alterations of cortical density in some higher olfactory processing centers, but unchanged cortical thickness [29]. Moreover, there were no volumetric differences in the dorsal stream structures between congenitally blind and controls [27]. The perception modality was not better differentiated in primary sensory cortices in the blind than in the sighted group [62]. Also, early visual deprivation may lead to functional neuroplasticity earlier than structural neuroplasticity in young blind patients. It seems that the brain of individuals who are born blind undergoes substantial reorganization compared to that of sighted individuals [27]. Regarding the hearing system, a lack of early visual experience affects the ability to encode the surrounding space, and congenital blindness influences the processing and retrieval of spatial auditory items [20].

It has been also shown that visual deprivation affects not only cortical plasticity but also the functional architecture of the cortex. There is evidence of cross-modal activation of higher visual areas in blind participants, including the representation of specific imagined auditory features in visual area V4 [62]. We can presume that sustained changes in behavior are associated with changes in cortical representations.

These novel findings were possible owing to advancements in MRI technology. Exploring IRD, with known molecular mechanisms and with genetically modified animals [63], seems to be one of the best models to test and find evidence of brain neuroplasticity. Increasingly high resolutions of nervous tissue imaging obtained through the use of a high level of magnetism allow the examination of even very small anatomical structures of the central nervous system that were previously elusive. Modern types of encephalography also support new discoveries related to neuroadaptations [64]. Currently, these examples [28] provide arguably the best available information about neuroadaptations in response to the loss of one or two senses. Our review has also shown some limitations of behavioral tests [65]. Furthermore, based on the range of available data, our review has revealed a trend in research looking for mechanisms that could be influenced to increase brain neuroplasticity [66,67]. 

The human cortex is made up of specialized regions that serve different functions, such as visual motion perception and language processing. How do genes and experience contribute to this specialization? Studies of plasticity show that cortical areas can change function from one sensory modality to another. There are studies that demonstrate that input during development can alter cortical function even more dramatically. In blindness, a subset of “visual” areas becomes specialized for language processing. These data suggest that the human cortex has a broad functional capacity during development and that input plays a major role in determining functional specialization [68]. In blindness, “visual” cortices are active during auditory and tactile tasks [69,70]. The precise cognitive and anatomical nature of these effects remains uncertain. There is growing research evidence to show that “visual” cortices of blind individuals respond to linguistic stimuli [48,71,72,73,74]. The endpoint of plasticity, however, is not always beneficial and can lead to significant maladaptive outcomes depending on the nature and extent of the neuropathogenic process, the stage of neurodevelopment at which it occurs, as well as the integrity of homeostatic regulatory mechanisms [75].

The data we have collected indicate that retinal diseases do not manifest locally. Progressive, inherited disorders of the retina directly affect other elements of the central nervous system [11,28]. The rare eye congenital diseases leading to blindness are a highly heterogeneous group of disorders, and it is difficult to make relationships related to particular disease entities. The loss of one of the senses, depending on the cause, significantly changes the architecture of neural connections [55,76]. This is indicated as a conclusion of the results of behavioral tests that scientists have shown for decades (loss of one of the senses “sharpens” the other) [44,71,72]. Although there is currently no cure for the majority of retinal dystrophies, recent treatments have focused on restoring visual function via a variety of approaches, such as gene augmentation therapy or retinal implants. However, the success of retinal restoration depends on the extent to which the rest of the visual pathway function and structure have remained stable from the point that sight was lost, without any significant degeneration or reorganization. 

We can speculate that in the future, MRI will likely play an important role in assessing the impact of eye disease on the visual pathway and how it progresses over time. The results may provide a new tool to assess the ability of IRD patients to adapt to altered visual inputs. We can indicate whether the visual cortex reorganizes its function and retinotopy after early retinal deficit. Structural MRI studies in IRD patients can contribute to investigating whether there is brain degeneration in eye diseases, determine optimal timing for retinal implant insertion, and establish structural MRI examination as a diagnostic tool in ophthalmology. Moreover, we can prove that the adult brain has sufficient short-term plasticity to benefit from prospective therapies in IRD. Additionally, functional MRI might be a tool for assessing visual sparing as it is potentially more feasible and sensitive than psychophysical or ophthalmological testing.

## 5. Conclusions

The use of neuroimaging allows us to visualize changes in structures in various parts of the brain that occur under the influence of retinal diseases. Small structures of the central nervous system, for example, LGN, previously impossible to visualize using traditional MRI, became possible to examine thanks to the use of a higher electromagnetic field and additional tools in new types of MRI. Therefore, high magnetic field MRI has become the best tool for exploring the impact that various retinal diseases have on each element of the brain. Knowledge of the pathogenesis of retinal disease and its overall impact on the CNS may become the basis for the construction of new possible therapeutic strategies in the future. 

## Figures and Tables

**Table 1:** Summary of reviewed studies.

Order of Appearance in PubMed	Reference Number	Type of the Study/Used Methodology	Studied Group	Aim of the Study	Main Results and Conclusions
1	Hofstetter et al., 2019 [48]	Neuroimaging/MRI Diffusion tensor imaging (DTI)	Blind retinitis pigmentosa (RP) patients (RP-BL) (*n* = 10) RP patients with tunnel vision (*n* = 10)	To study the progression of white matter plasticity in a state of deteriorating visual field perception.	The results demonstrated gradual changes in diffusivity that are indicative of degenerative processes in the primary visual pathway comprising the optic tract and the optic radiation. This reorganization may point to microstructural plasticity underlying adaptive behavior and cross-modal integration after partial visual deprivation.
2	Zhou et al., 2019 [25]	Neuroimaging/MRI Behavioral (training with a visual-to-tactile sensory substitution system)	Congenitally blind (CB) (*n* = 12)	To correlate the performance of congenitally blind individuals (CB) with the size of brain structures.	There are no volumetric differences in the dorsal stream structures between CB and controls; this brain network is likely to be recruited in both groups.
3	Rączy et al., 2019 [60]	Neuroimaging/MRI (and individual in-ear recordings)	EB (early-blind) participants (*n* = 16)	To functionally and structurally investigate how early blindness affects brain networks involved in spatial hearing.	10
4	de Borst and de Gelder, 2019 [61]	Neuroimaging/MRI (fMRI scans before and after 2-h behavioral training) Behavioral/training using an auditory script based on a visual-to-auditory sensory-substitution-device (SSD)	Congenitally blind (*n* = 11)	To characterize the temporal dynamics of computation-specific neural specialization in the deprived visual cortex when processing an atypical sensory input	Observations after 2 h of SSD training indicate the recruitment of the deprived ventral visual stream by auditory stimuli; computation-selective cross-modal recruitment requires longer training to establish.
5	Rimmele et al., 2019 [23]	Electroencephalography and Behavioral (to detect rare pseudowords)	Congenitally blind individuals (*n* = 8)	To investigate that spatial selective attention is necessary for the processing of affective prosodies after visual deprivation from birth	Blind individuals were more efficient in detecting deviant syllables at the attended loudspeaker and had higher auditory N1 amplitude than controls. The results provide evidence for “emotion-general“ auditory spatial selective attention effects in congenitally blind individuals and suggest a potential reorganization of the voice processing brain system following visual deprivation from birth.
6	Chebat et al., 2020 [27]	Neuroimaging/MRI and Magnetoencephalography/	Congenital blind (CB) (*n* = 26)	To characterize alterations in spectral profiles in CB in order to map visual deprivation-related spectral changes to areas in the brain.	The results suggest that the power increases in the theta-to-beta frequency bands in auditory and frontal brain regions may reflect adaptive sensory or higher cognitive processing in blind individuals, while altered spectral profiles in visual brain regions (a lower alpha and a gamma peak) may indicate a change in the excitation–inhibition balance.
7	Cavaliere et al., 2020 [62]	Neuroimaging/MRI	Early-blind adolescents (EBAs) (*n* = 23)	To investigate the effects of residual light perception on brain microstructure and function in EBAs.	The study demonstrated significant microstructural and functional alterations in EBAs with and without residual light perception. These findings provide additional evidence that early visual deprivation may lead to functional neuroplasticity earlier than structural neuroplasticity in EBAs.
8	Topalidis et al., 2020 [19]	Neuroimaging/MRI (fMRI scans during behavioral tasks) Behavioral/(3D picture and voice training)	Congenitally blind (*n* = 8)	To show that auditory versus tactile perception evokes similar intra-modal discriminative patterns in congenitally blind compared to sighted participants	The analyses showed that classification accuracies were significantly lower for the blind group in the primary motor cortex area 4p, for primary auditory cortex areas Te1.0 and Te1.1, primary 11somatosensory cortex areas 1, 2, and 3b, and primary motor cortex area 4a. This indicates that perception modality was not better differentiated in primary sensory cortices in the blind than in the sighted participants.
9	Lubinus et al., 2021 [26]	Magnetoencephalography/ Behavioral: memory training with (1) voices; (2) tactile motion stimuli; (3) an active training-control task.	Congenitally blind (*n* = 27)	To study the cross-modal reorganization of the neuronal mechanisms underlying the recruitment of the visual cortex for non-visual tasks in congenitally blind participants	In blind participants, beta-band networks largely emerged during the training, and connectivity increased between brain areas involved in auditory working memory and, as predicted, the visual cortex. These findings highlight long-range connectivity as a key mechanism of functional reorganization following congenital blindness and provide new insights into the spectral characteristics of functional network connectivity.
10	Ptito et al., 2021 [58]	Neuroimaging/MRI	Congenitally blind (CB) (*n* = 22) late-blind (LB) (*n* = 14)	To measure the integrity of the retino-fugal system using structural MRI images	The optic nerve, optic tract, optic chiasm, and lateral geniculate nucleus (LGN) were reduced by 50 to 60% in CB and LB. There were no differences between CB and LB. In LB, optic nerve volume correlated negatively with blindness duration.
11	Lin et al., 2022 [24]	Neuroimaging/MRI +Behavioral/verb generation and nonword reading	Congenitally blind: children (*n* = 8); adults (*n* = 22)	To investigate how the deprivation of congenital visual sensory information modulates the development of the thalamocortical network	Blind children had markedly lower (gray matter volume (GMV) values in the “visual” thalamic regions than sighted children.
12	Cappagli et al., 2022 [22]	Behavioral/localize positive and negative numbers in space	Children with low vision (*n* = 3) Children with complete blindness (*n* = 8)	To investigate how congenital visual deprivation affects the ability to represent positive and negative numbers in horizontal and sagittal planes in visually impaired children	Long-term visual deprivation alters the ability to identify the spatial position of numbers independently of the spatial plane and the number polarity, suggesting that visual experience might have a differential role in numerical processing depending on number polarity.
13	Guerreiro et al., 2022 [18]	Neuroimaging/MRI	Patients treated for bilateral congenital cataracts (*n* = 9)	To explore whether early visual deprivation may affect the extent to which typically visual, motion-selective area hMT responds to moving visual stimuli	The results suggest a significantly attenuated functional selectivity of area hMT for visual motion processing in cataract-reversal individuals, consistent with the notion that visual cortical specialization depends on early visual experience.
14	Setti et al., 2022 [20]	Behavioral/acoustic simulation: recalling sequences of spatialized auditory items in the same or reverse order	Congenitally blind (*n* = 9)	To investigate how spatial working memory skills as well as the processing and retrieval of distal auditory spatial information are influenced by visual experience	Blind participants had a shorter memory span in the backward than the forward order of presentation. A lack of early visual experience affects the ability to encode the surrounding space.
15	Zohary E et al., 2022 [37]	Behavioral/gaze-cueing paradigm in at least one of two experiments before and after cataract surgery	Congenital cataract + late surgery (*n* = 19) Congenital cataract + early surgery (*n* = 11)	To check if gaze understanding can be learned later if vision is extremely poor throughout early childhood	Late-operated groups failed to show eye gaze-following effects and fixated less than controls on the eyes—two spontaneous behaviors typically seen in controls. The restored vision Is effective for the head, but not the eye, confirming that gaze-following mechanisms based on eye position information develop normally despite a brief deprivation period in early development (typically 4 to 6 mo).
16	Battal et al., 2022 [59]	Neuroimaging/MRI	Congenitally blind (CB) (*n* = 12) and late-blind (LB) (*n* = 15)	To investigate the effects of blindness on the structural and functional integrity of the corpus callosum and the anterior commissure (AC)	The results show a larger anterior commissure (AC) for CB, decreased fractional anisotropy (FA) in the posterior part of AC (pAC), and selective reduction of the splenium of the corpus callosum (CC) in CB and LB. The results further support the findings that the splenium, a structure primarily composed of fibers connecting the visual areas of the brain, is indeed sensitive to visual deprivation in both CB and LB.
17	Chouinard-Leclaire et al., 2022 [29]	Neuroimaging/MRI	Congenitally blind (*n* = 16)	To investigate whether congenitally blind (CB) individuals show brain alterations in the olfactory system by comparing cortical morphology and olfactory bulb (OB) volume	The results showed that CB individuals exhibited smaller OB and alterations of cortical density in some higher olfactory processing centers, but unchanged cortical thickness. These findings suggest that a lifelong absence of visual input leads to morphological alterations in olfactory processing areas.
18	Arbel et al., 2023 [28]	Neuroimaging/MRI	Congenitally blind (*n* = 15)	To investigate which areas deprived of stimuli of the brain expand, preserve their function and to what extent they acquire new ones	The results reveal a double dissociation, with tactile orthographic priming in the vOT (ventral occipitotemporal cortex) and auditory priming in general language areas/vOT in the blind group serving multiple functions, one of which, orthographic processing, overlaps with its function in the sighted group.

## Data Availability

Not applicable.
